# Using Attentional Bias Modification as a Cognitive Vaccine Against Depression

**DOI:** 10.1016/j.biopsych.2012.04.014

**Published:** 2012-10-01

**Authors:** Michael Browning, Emily A. Holmes, Matthew Charles, Philip J. Cowen, Catherine J. Harmer

**Affiliations:** a Department of Psychiatry, University of Oxford, Oxford, United Kingdom; b Research Department of Clinical, Educational and Health Psychology, University College London, United Kingdom

**Keywords:** Attention, cognitive bias, depression, experimental medicine, prevention

## Abstract

**Background:**

Negative attentional biases are thought to increase the risk of recurrence in depression, suggesting that reduction of such biases may be a plausible strategy in the secondary prevention of the illness. However, no previous study has tested whether reducing negative attentional bias causally affects risk factors for depressive recurrence. The current experimental medicine study reports the effects of a computerized attentional bias modification (ABM) procedure on intermediate measures of the risk of depressive recurrence (residual depressive symptoms and the cortisol awakening response) in patients with recurrent depression.

**Methods:**

Sixty-one patients with at least two previous episodes of depression who were currently in remission were randomized to receive either an active (positive) or placebo computer-based ABM regime. The ABM regime presented either pictures of faces or words. Residual depressive symptoms, measured using the Beck Depression Inventory and the cortisol awakening response were measured immediately before and after completion of the bias modification and then again after 4 weeks' follow-up.

**Results:**

Positive, face-based ABM reduced both measures of recurrence risk (Beck Depression Inventory and cortisol awakening response). This effect occurred during the month following completion of bias modification. Word-based modification did not influence the outcome measures.

**Conclusions:**

Positive face-based ABM was able to reduce intermediate measures of recurrence risk in previously depressed patients. These results suggest that ABM may provide a “cognitive vaccine” against depression and offer a useful strategy in the secondary prevention of the illness.

Depression is overwhelmingly a recurrent disorder with 80% of patients experiencing more than one episode in their lifetime (1). Secondary preventative strategies that aim to lower the risk of recurrence in previously depressed patients are therefore likely to be a particularly important method of reducing illness burden.

The highly recurrent nature of depression indicates that certain individuals possess vulnerability factors that predispose them to repeatedly develop acute episodes of the disorder. Preventative treatment strategies are predicated on identifying and ameliorating these vulnerability factors (2) because doing so will reduce an individual's risk of subsequent acute episodes. Previously identified candidate vulnerability factors for recurrence in depression include clinical characteristics such as the persistence of subsyndromal symptoms following treatment (3), social factors (4,5), and measures of endocrine and neurocognitive functioning such as an exaggerated cortisol awakening response (CAR) (6) and the presence of negative cognitive biases (7). The current study focuses on negative cognitive biases and examines whether they may be a useful target in the secondary prevention of depression.

Depressed patients tend to pay attention to, interpret, and remember information in a negative manner (7). Importantly, however, these negative cognitive biases are found not only in currently depressed patients but are also present in nondepressed individuals who are at high risk of developing depression. This indicates that negative cognitive biases may be true vulnerability factors for depression rather than simple markers of mood. For example, depressed patients tend to have difficulty redirecting their attention (8) away from negative, relative to positive, stimuli (9,10) with a similar pattern being found in previously depressed, currently euthymic individuals (11) and in never-depressed individuals who are at high risk because of a family history of the illness (12). Thus, there is evidence that negative attentional biases are associated not just with current depressive symptoms but also with the risk of developing depression. However, for a risk factor to be considered a target for preventative interventions, there should be a direct causal relationship, rather than simply an association, between it and the likelihood of recurrence. Recent experimental work has demonstrated that attentional biases may be trained using computerized attentional bias modification (ABM) tasks (13–15). These deceptively simple tasks (Figure 1C) alter attentional bias by placing a probe, to which the patient has to respond, behind either a positive or negative stimulus. If the probe always appears in the location of the positive stimulus, a habit of automatically directing attention toward positive stimuli is encouraged—that is, patients develop a positive attentional bias. This manipulation may be compared to a neutral control condition in which the probe appears equally often behind the positive and negative stimuli and that therefore does not alter attentional bias. In this study, we used a randomized experimental design to test whether ABM causally influences markers of relapse risk in depression. In other words, we assess whether ABM may be used as a “cognitive vaccine” (16) in individuals at high risk of recurrence.

We used an experimental medicine approach to test whether modification of negative attentional bias in previously depressed patients was able to influence known risk factors for depressive recurrence. Currently euthymic, previously depressed patients were randomly assigned to receive either an active (positive) or placebo (neutral) ABM regime, which was completed over 2 weeks. The impact of the bias modification regime on two measures of vulnerability to depressive recurrence, residual depressive symptoms (3) and CAR (6), was assessed before and after completion of the regime and then again after 1 month follow-up. The study also examined the most effective method for delivering the bias modification. Previous work using ABM in other populations has employed either word or face stimuli in the tasks (13). Although verbal stimuli may more closely match the abstract ruminative processing style characteristic of depression (17), visual face stimuli are thought to elicit a more profound emotional response (18) and therefore may result in a greater bias modification effect. Patients were therefore additionally randomized to receive bias modification with either a word- or face-based task (Figure 1B).

We predicted that positive, relative to placebo, ABM would lead to a reduction in the markers of recurrence risk (residual depressive symptoms and CAR). Positive attentional bias is not thought to alter mood directly but rather to protect against the negative effects of stressful environmental interactions (7); it was therefore predicted that the effect of ABM on mood would accumulate with exposure to such interactions over time—that is, residual depressive symptoms and CAR would decrease across the bias modification and follow-up period. Because no previous study has compared word- and face-based ABM in this population, we had no strong rationale for predicting whether one form would be more effective than the other.

## Methods and Materials

### Patients

Sixty-one patients with recurrent depression who were not currently depressed were recruited from the community by advertisement. The inclusion criteria were two or more episodes of lifetime depression with no episodes occurring within the previous 6 months. Diagnosis of lifetime depression (as well as any comorbid diagnoses) and absence of current depression was confirmed during a screening visit in which the Structured Clinical Interview for DSM-IV Clinical Version (SCID-CV) (19) was administered by a trained interviewer. Exclusion criteria were age less than 18 or more than 65 years, use of any psychotropic medication or psychological therapy within the previous 3 months, and a current or lifetime psychotic disorder or current symptomatology (e.g., suicidality) that was deemed to require immediate treatment. Basic demographic and clinical information, including an estimate of verbal IQ (National Adult Reading Scale; NART) (20) was collected at the screening session and is summarized in Table 1. Written informed consent was obtained from all patients before enrollment into the study, which had received approval from the local research ethics committee. Full data were available on all but one participant who did not attend the final assessment session. Data analysis was carried out on all available data (i.e., 61 patients for the first two assessment sessions and 60 patients for the final session).

### Procedure

**Figure 1:**
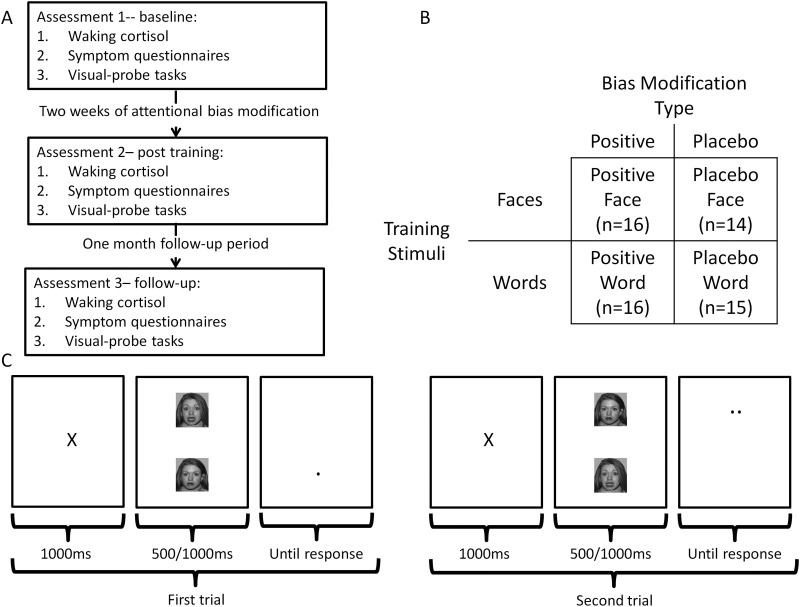
Study design and attentional bias modification (ABM) task used. **(A)** Patients completed three assessment sessions, immediately before and after 2 weeks of ABM and then again a month later. The assessment measures completed during both sessions are listed. **(B)** Each participant was randomly assigned to one of four treatment groups using a factorial design. This design allows assessment of the main effects of both ABM type and the stimuli used in ABM as well any interaction between the two. **(C)** Two example trials from the ABM task completed by patients. On each trial two stimuli were presented, followed by a probe (one or two dots) to which the patients had to respond. During positive ABM (shown) the probe appeared behind the more positive of the two stimuli; the placebo ABM condition was identical in every respect other than that the probe was equally likely to appear behind either stimulus. The stimuli used during ABM were either faces (shown) or words.

The study procedure is summarized in Figure 1A. Following enrollment in the study, patients were randomized (with stratification by gender) into one of four groups (Figure 1B). Group membership (positive face ABM, placebo face ABM, positive word ABM, placebo word ABM) determined the form of ABM undertaken. ABM was completed twice daily over the course of 14 days (28 sessions total). Patients were informed that the study examined how “thinking style changed over time and how this is related to mood”; the specific rationale underlying the ABM was not explained, and patients were blind to group allocation. Data were collected in three assessment sessions, the first immediately before ABM commenced, the second immediately after the 2 weeks of ABM, and the last 1 month following this. On the morning of each assessment session, patients collected waking salivary samples for analysis of cortisol, and during the assessment sessions, self-report symptom measures and tasks assessing attentional bias were collected. The bias assessment tasks provide a check that the ABM regime was having the expected impact on attentional function.

### ABM Task

The ABM task used (Figure 1C) was a computerized, visual-probe bias modification procedure (21) that was developed to alter attentional bias to emotional information. Similar tasks have been found to improve symptoms of current anxiety and depression (13,22). On the basis of the widely used visual-probe task (23), a pair of stimuli were briefly presented and followed by a probe (one or two dots), which appeared behind one of the stimuli. Participants were required to press one of two buttons to indicate the number of dots in the probe. The type of stimuli used during the ABM task were either pictures of faces or words and were selected to have positive, neutral, or negative valence with each trial of the task displaying stimuli from two valences (vertical visual angle between the center of stimuli ≈12°). This resulted in three possible stimuli pair types: positive-neutral, positive-negative, and negative-neutral. During the positive ABM condition, the probes replaced the relatively positive stimuli of a given pair and thus, when completing the positive ABM, patients learn to deploy their attention toward positive stimuli as they predict the probe location—that is, they develop a positive attentional bias. Placebo ABM was identical in every respect other than the location of the probe, which was equally often found behind the positive and negative stimuli. Further details concerning the ABM task and stimuli selection are provided in Supplement 1.

### Measures of Residual Symptoms

Residual symptoms were measured at all three assessment sessions using standardized questionnaires of self reported depressive (Beck Depression Inventory; BDI) (24) and anxious (Trait subscale of the Spielberger State-Trait Anxiety Inventory) (25) symptoms. These measures were supplemented by an observer reported measure of depressive symptoms, the Hamilton Rating Scale for Depression (HRSD) (26), which was collected by a trained administrator who was blind to participant group allocation.

### Measures of Waking Cortisol

Patients collected their own saliva samples upon waking on the morning of each assessment session. The patients were carefully instructed to take the first saliva sample as soon as they awoke and to take four additional samples at 15-minute intervals. During the sampling, the subjects were asked not to eat or drink. Saliva samples were collected by using a salivette device (Sarstedt, Leicester, United Kingdom) in which saliva is absorbed into a cotton roll and then expressed into a sterile vial. The CAR was defined as the difference between the cortisol level on waking and the highest level achieved in the following 4 samples (27). See Supplement 1 for details of the cortisol assay used.

### Measure of Attentional Bias

Standard visual-probe tasks were used to assess attentional bias. These tasks were identical to the ABM task with the exception that the location of the probe was random for all patients (allowing assessment of attentional bias). Regardless of the type of stimuli used in the ABM task, all patients completed separate word- and face-based visual-probe tasks during each assessment visit. A measure of attentional bias toward the relatively more positive stimulus was calculated by subtracting the average reaction time to respond to trials in which the probe replaced the positive stimulus from those in which the probe replaced the relatively negative stimulus.

### Statistical Analysis and Data Reduction

Analysis was performed using split plot analyses of variance (ANOVA) with the between subject factors of ABM type (positive vs. placebo) and ABM stimuli (words vs. faces). Time (before ABM, after ABM, follow-up) was included as a within-subject factor. Where assumptions of equality of variance were not met, the Huynh-Feldt correction was used, although unadjusted degrees of freedom are reported for clarity. For the visual probe data, mean reaction times were used in the analysis. These were calculated after removing error trials and extreme responses, which were defined as those which lay outside 200–1200 msec or which were greater than three standard deviations from an individual's mean (extreme outliers were removed before calculating individual means and standard deviations because these measures are particularly sensitive to the effects of outliers; however, the results from the visual probe task were identical if outliers were left in and median reaction times were used). Three patients had extreme levels of cortisol excretion (i.e., >3 SD from the group mean) and were not included in the analysis.

## Results

### Group Demographics, Baseline Measures, and Compliance

As can be seen from Table 1, the groups were well matched on baseline demographics, IQ, and measures of illness severity. Patients had been depressed on an average of three previous occasions. Compliance was generally high with only eight patients completing fewer than 25 sessions of ABM and no difference in compliance between the groups.

### The Effect of ABM on Residual Symptoms

**Figure 2:**
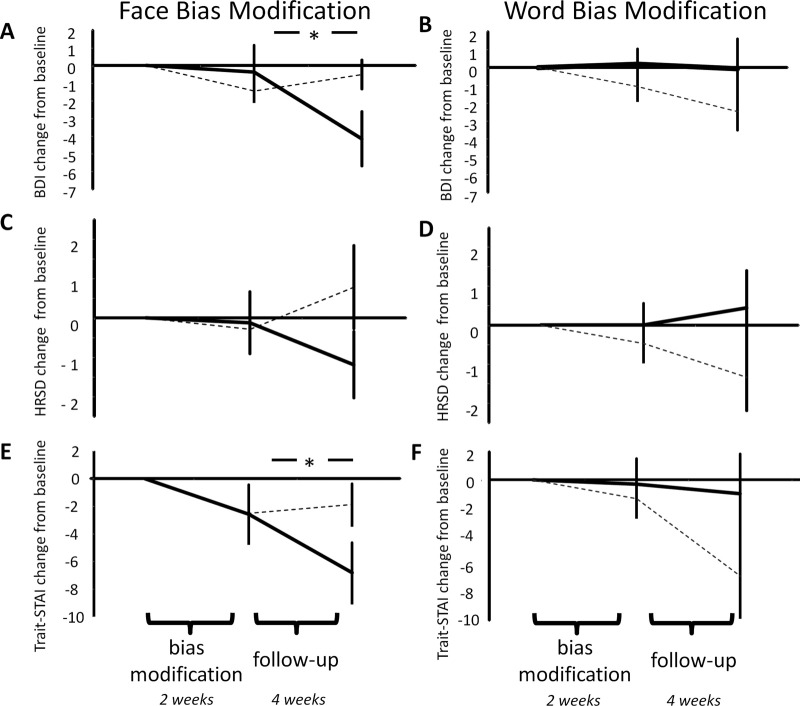
The effects of attentional bias modification (ABM) on residual symptoms of depression measured using the BDI **(A, B)** and the HRSD **(C, D)** and on symptoms of anxiety measured using the trait-STAI **(E, F)**. Symptoms, which are displayed as a change from baseline of the mean scores, were measured at three time points; before bias modification, after bias modification, and after 1-month follow-up. The symptoms of both depression and anxiety were significantly altered by face- but not word-based ABM. The effect of ABM occurred during follow-up with no difference in groups seen during the bias modification period. Solid line, positive ABM; dashed line, placebo ABM. Error bars represent SEM. *p < .05 for post hoc test of interaction. BDI, Beck Depression Inventory; HRSD, Hamilton Rating Scale for Depression; Trait-STAI, the trait subscale of the Spielberger State-Trait Anxiety Inventory.

Positive, relative to placebo, ABM influenced the residual depressive symptoms reported by patients using the BDI, but this effect depended on whether the ABM used faces or words [ABM type × ABM stimuli × time; *F*(2,112) = 3.7, *p* = .03]. There was no general ABM type × time effect [*F*(2,112) = 1.1, *p* = .35]. As can be seen from Figure 2 positive, face-based ABM lead to a reduction of symptoms compared with placebo ABM [Figure 2A; *F*(2,56) = 3.7, *p* = .03], whereas word based ABM had no significant effect [Figure 2B; *F*(2,56) < 1, *p* = .4]. Interestingly, the beneficial effect of positive face ABM displayed a time lag; symptoms did not change across the 2 weeks of ABM [*F*(1,28) < 1, *p* = .5]; rather, they reduced during the follow-up period [*F*(1,28) = 5.9, *p* = .02] with this change resulting from a significant drop in symptoms reported by the positive face ABM group [*t*(15) = 2.4, *p* = .03] and no significant change seen in the placebo face ABM group [*t*(13) < 1, *p* = .4].

Observer reported depressive symptoms, measured using the HRSD, revealed an identical ABM type × ABM stimuli × time interaction [Figure 2C and 2D; *F*(2,112) = 3.4, *p* = .04]; however, in this case, the post hoc tests of the interaction for face training was significant only at a trend level [*F*(1,28) = 2.9, *p* = .09].

Symptoms of anxiety, measured using the trait subscale of the STAI displayed the same specific effect of positive face ABM as that seen for the BDI [Figures 2E, F; *F*(2,112) = 3.3, *p* = .05] with the beneficial effect again occurring during follow-up [*F*(1,28) = 5.2, *p* = .03] and being driven by a significant reduction of anxious symptoms in the positive face ABM group [*t*(15) = 2.4, *p* = .03].

### The Effect of ABM on the CAR

**Figure 3:**
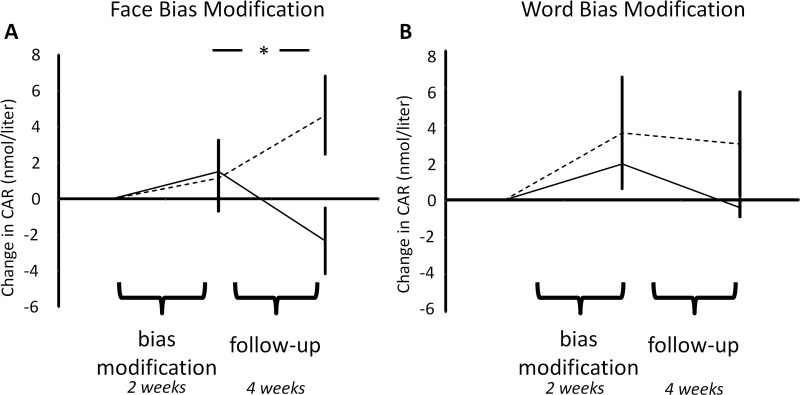
The effects of attentional bias modification (ABM) on cortisol awakening response (CAR). Results display the change from baseline of the mean CAR measured before bias modification, after bias modification, and after 1-month follow-up. Face-based ABM **(A)** produced a significant effect on CAR, whereas word-based ABM **(B)** had no effect. Again, the effect of face ABM was seen during the follow-up period. Solid line, positive ABM; dashed line, placebo ABM. Error bars represent standard error of the mean. *p < .05 for post hoc test of interaction.

Analysis of the CAR response revealed a significant ABM type × ABM stimuli interaction [*F*(1,53) = 4.3, *p* = .04], which was present across the three time points [ABM type × ABM stimuli × time; *F*(2,106)<1, *p* = .4]. In keeping with the effects on residual symptoms, face-based ABM influenced the CAR [Figure 3A; *F*(2,54) = 4, *p* = .02], whereas word-based ABM did not [Figure 3B; *F*(2,52) < 1, *p* = .8]. Specifically, positive relative to placebo face ABM caused a reduction of the CAR across the follow-up period [*F*(1,27) = 5, *p* = .03] with no change being seen during ABM [*F*(1,27) < 1, *p* = .9]. This relative reduction was produced by a nonsignificant decrease in the positive ABM group during follow-up [*t*(14) = 1.7, *p* = .1] and a nonsignificant increase in the placebo group [*t*(13) = 1.5, *p* = .2].

### The Effect of ABM on Attentional Function

**Figure 4:**
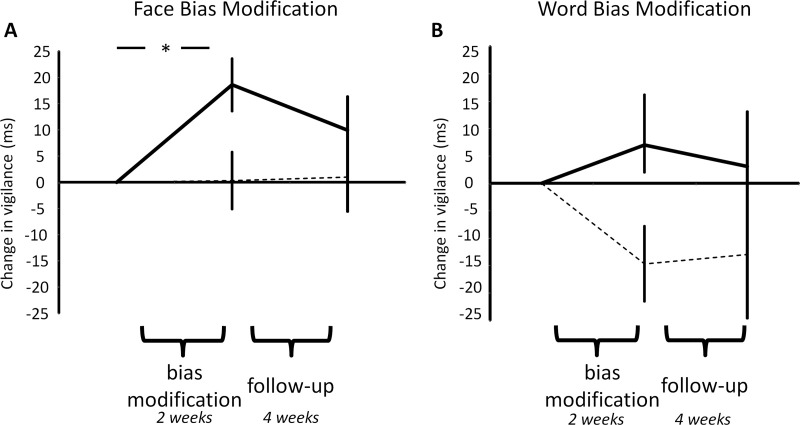
The effects of attentional bias modification (ABM) on attentional vigilance to word stimuli measured using the visual probe task. Vigilance is calculated so that a greater positive number represents increased vigilance for the positive stimulus, whereas a negative number represents vigilance for the negative stimulus. Results display the change from baseline of the mean attentional bias measured before bias modification, after bias modification, and after 1-month follow-up. Positive face-based ABM **(A)** produced a significant increase in vigilance toward the positive stimuli, whereas placebo word-based ABM **(B)** resulted in a trend-level decrease in vigilance. Unlike the measures of recurrence risk (Figures 2 and 3), the effect of ABM was seen during the bias modification period. Solid line, positive ABM; dashed line, placebo ABM. Error bars represent standard error of the mean. *p < .05 for post hoc test.

Positive relative to placebo bias modification produced a differential effect on attentional bias (Figure 4), as measured by the word based visual-probe task [ABM type × time; *F*(2,112) = 3.1, *p* = .05]. However, unlike the previous results, this effect did not significantly differ between the face- and word-trained groups [ABM type × ABM stimuli × time; *F*(2,112) < 1, *p* = .9]. The effect of ABM was seen across the bias modification period [*F*(1,57) = 8.3, *p* = .006] with no significant change occurring across follow-up [*F*(1,56) < 1, *p* = .4]. When considering the within group effects produced, positive-face ABM led to a significant increase in positive bias across the modification period [*t*(15) = 3.7, *p* = .002], whereas face placebo ABM did not change the bias [*t*(13) < 1, *p* = .9]. Interestingly, the effect of word ABM was driven by a trend level decrease in bias in the placebo word group [*t*(14) = 2.1, *p* = .051] with no effect seen for positive word ABM [*t*(15)<1, *p* = .5]. The correlation between change in attentional bias across the bias modification period and reduction in BDI or CAR across the follow-up period in the positive face-ABM group was not significant [*r*(16) = .37, not significant, and *r*(15) = .34, not significant, respectively]. Additional analysis is provide in Supplement 1.

Attentional bias, assessed using face stimuli, was insensitive to the effect of the ABM interventions [*F*(2,112)<1, *p* = 1]. This remained the case when analysis was restricted to those who had completed face based ABM [*F*(2,56) < 1, *p* = .7].

## Discussion

In this study, an experimental medicine model was used to test the prediction that alteration of attentional bias using a simple computerized bias modification task would causally influence markers of the risk of depressive recurrence. The results indicate that positive ABM, when administered using pictures of faces, was able to reduce two measures of risk of depressive recurrence: residual depressive symptoms as measured by the BDI and the CAR. Reminiscent of the delay in onset of therapeutic effect reported for antidepressant medications (28), the benefit of face-based ABM was found to lag behind its administration. In contrast, when words were used in the ABM task, no significant effect on recurrence risk was seen. These results support the proposal that modification of attentional bias, at least when achieved using faces, may be used as a cognitive vaccine (16) in the secondary prevention of depression.

Cognitive models (7) of depression have suggested that negative attentional biases are causally related to recurrence risk. Consistent with this, previous laboratory-based studies have demonstrated negative attentional biases in populations with a heightened risk of developing further depressive episodes (11,12). However, cross-sectional studies such as these leave open the possibility that negative attentional biases are markers of depressive risk rather than causally influencing that risk. The finding that modifying the biases produces change in other measures of recurrence risk reported here provides the first experimental evidence supporting a causal role for these negative biases in the risk of recurrence. These results are consistent with a growing recent literature suggesting that modifying biases directly through computerized tasks can be beneficial for psychopathology in anxiety (13) and with an emerging literature suggesting that such techniques may be used in depression (29).

Secondary prevention is recognized as a key goal in the long-term management of depression (28). The current study provides initial evidence for a novel method of achieving this goal. Specifically, it predicts that using face-based positive ABM will reduce the likelihood of developing further episodes of depression. Ultimately these results, which used intermediate markers of risk of recurrence, must be confirmed in large-scale trials in which patients are followed up for a sufficient period of time to allow an effect on clinical recurrence rates to become apparent. However, the experimental medicine approach used in this study provides an efficient means of initially assessing and refining novel treatments before committing to large-scale clinical trials (30). The success of the face- rather than word-based ABM demonstrated in this study is an example of the insights this approach can provide. Whereas positive face-based ABM led to an increase in positive attentional bias as well as improvement in both residual symptoms and CAR, positive word-based ABM had none of these effects. The differential impact of the two forms of ABM may reflect the greater emotional impact of the biologically prepared faces when compared with the more abstract verbal stimuli (18). Regardless of the specific reason for the difference in efficacy, the value of the current study is in suggesting that future trials of ABM in depression would be wise to use face stimuli.

A number of previous studies have linked both residual depressive symptoms and the function of the hypothalamic-pituitary-adrenal axis to recurrence risk in depression. Conceptually the association between persistence of symptoms following treatment and subsequent recurrence is particularly straightforward and, indeed, a number of prospective clinical studies have demonstrated significantly increased recurrence rates in patients with such symptoms (e.g., from 25% to 76%) (31,32). Although the measure of hypothalamic-pituitary-adrenal axis dysfunction used in our study, the CAR, suggests a possible mechanism through which the risk of recurrence may be raised, the clinical evidence linking raised CAR and depressive recurrence is somewhat less developed than for residual symptoms because it has never been used, to our knowledge, to directly predict recurrence rates in longitudinal studies. However, cross-sectional studies provide indirect evidence of such a link by demonstrating that CAR is raised not only in patients who are depressed (33) but also in nondepressed participants who are at high risk of recurrence or illness onset because of a personal (34) or family (35) history of the illness.

A related question concerns the precise mechanism through which ABM was able to alter depressive symptoms in the current study. Previous authors have suggested that negative attentional biases may alter depressive symptoms via an effect on ruminative processes (36). Our results raise an additional possibility: that the effect of ABM was mediated via the changes in CAR—that is, positive face-ABM reduced CAR response, which in turn reduced depressive symptoms. However, the sample sizes used in the current study were not sufficient to support the formal mediation analysis, which would be required to test this proposal. Furthermore, no significant correlations were found between change in attentional bias during ABM and change in either BDI or CAR during follow-up in the positive face ABM group. Thus, although the current study was able to demonstrate a causal influence of ABM on both CAR and residual symptoms, the precise mechanisms underlying these findings remain obscure.

These results may be relevant to understanding the mechanisms of complex psychotherapies. A number of these psychotherapies, such as cognitive behavioral therapy (37) and mindfulness therapy (38), have been associated with reduced recurrence rates in depression. It has been suggested that both of these forms of therapy work, at least in part, by altering attentional function (39,40). The ABM used in the current study may be viewed as a distillation of one component of these more complex therapies (14,41,42) and suggests that the affective attentional bias components of the therapies may account for some of their effect on recurrence rates.

Some limitations of the study should be acknowledged. First, to ensure that patients were not currently depressed, we required a period of at least 6 months since the resolution of the last episode of depression. However, it has been estimated that up to 30% of patients will relapse within 3 months of recovery (43), and therefore our recruitment strategy may have biased our sample toward patients with a relatively favorable illness course. Consistent with this, baseline levels of depressive symptoms were relatively low, and the effect of ABM appeared more pronounced when symptoms were measured using a subjective (BDI) than objective (HRSD) rating. Second, the ABM task and collection of saliva samples was performed by the patients outside of a laboratory setting. It is likely that this aspect of the experimental design introduced a degree of variability in the performance of these tasks. However, we believe that the protocol used in the current study represents a pragmatic balance between the need for experimental precision and the burden this would place on patients. Third, the impact of ABM on attentional bias was measured using both face- and word-based visual probe tasks; however, the predicted effect of ABM was only seen when bias was measured with words. In a recent study, we found that a face-based visual probe task was sensitive to the effects of a similar ABM regime (30), so it seems unlikely that the task itself is insensitive. It is possible that the use of multiple manipulation check tasks (i.e., both word and face visual probe tasks) in the current study lead to participant fatigue and reduced the sensitivity of individual measures. Last, although 61 patients were recruited into the current study, the factorial design used meant that each group contained only a quarter of this number; it may therefore be that subtle effects of ABM, and in particular the effect of a word-based procedure, would be detectable if the sample size had been larger.

In this experimental study, positive face-based attentional bias modification was found to produce similar reductions in two intermediate risk markers for depressive recurrence: residual depressive symptoms and CAR. These results provide the first experimental evidence that attentional bias modification may form the basis of a useful approach in the secondary prevention of depression.

## Figures and Tables

**Table 1:** Participant Demographic and Clinical Information

	Positive ABM		Neutral ABM		
	Faces (*n* = 16)	Words (*n* = 16)	Faces (*n* = 14)	Words (*n* = 15)	*p*^a^
Age, Mean (SD), Years	34.6 (12.2)	40.9 (11.3)	37.8 (11.5)	40.9 (13.5)	.14
Sex, *n*, F:M	10:6	10:6	10:4	10:5	.59^b^
Years of Education, Mean (SD)	16.8 (3.8)	16.9 (4.0)	17.4 (3.0)	16.2 (1.7)	.43
VIQ (NART), Mean (SD)	114.6 (7.1)	114.6 (7.2)	117.6 (5.5)	117 (6.3)	.17
No. of Previous Episodes, Mean (SD)	3.6 (1.9)	3.1 (1.1)	3 (1.0)	3 (1.2)	.22
Total Illness Duration, Mean (SD), Months	22.3 (12.7)	31.9 (45.2)	32.8 (34.7)	21.6 (23)	.21
Time Since Last Illness Episode, Mean (SD), Months	22.3 (21.3)	41.6 (41.2)	48.6 (80.6)	38.4 (86.8)	.36
BDI Score, Mean (SD)	5.9 (6.9)	6.3 (5.4)	4.3 (3.7)	3.8 (4)	.14
Trait-STAI, Mean (SD)	45.3 (6.6)	44.4 (15)	44.1 (12)	43.9 (11.5)	.86
HRSD, Mean (SD)	3.1 (4.1)	2.9 (2.5)	1.8 (2.1)	3.1 (2.9)	.33
Compliance with ABM, *n*, Compliant:Noncompliant	14:2	14:2	13:1	12:3	.5^b^

ABM, attentional bias modification; BDI, Beck Depression Inventory; F, female; HRSD, Hamilton Rating Scale for Depression; M, male; NART, National Adult Reading Scale; Trait-STAI, the trait subscale of the Spielberger State-Trait Anxiety Inventory; VIQ, Verbal Intelligence Quotient.

a The *p* value reported is the lowest of the three comparisons: main effect of ABM stimuli, main effect of ABM type, or the stimuli × type interaction.

b Statistical analysis performed using logistic regression model; a univariate analysis of variance was performed in all other analyses.
