# Supplementary material for: Using Attentional Bias Modification as a Cognitive Vaccine Against Depression
Source: Biol Psychiatry. 2012 Oct 1;72(7):572–9. doi: 10.1016/j.biopsych.2012.04.014 (PMC3504298; doi:10.1016/j.biopsych.2012.04.014)
Supplement: Supplement 1 [file mmc1.pdf]

Using Attentional Bias Modification as a Cognitive Vaccine Against Depression

Supplemental Information

Supplementary Methods

Detailed Parameters of Attentional Bias Modification (ABM) Task

A single session of the task involved 96 trials with equal numbers of the three stimuli pair types. In addition, there were equal numbers of trials in which the stimuli were presented for 500 or 1000 ms before the probe was displayed (500 and 1000 ms trials were randomly presented throughout the course of the task). As with our previous work (1), more than one stimuli duration, and three valence categories were used to encourage generalization of the bias modification effect. All patients completed the ABM sessions twice daily at home (1) for two weeks using a laptop computer, which was supplied to them for the duration of the study.

Stimuli Selection. Face stimuli were taken from a range of previously published sources (2-5). Happy faces were used as positive stimuli, neutral faces as neutral stimuli and angry and fearful faces as negative stimuli. Positive, neutral and negative (including both physically and socially threatening) word stimuli were selected from a range of previously published sources (6-34). A total of 288 word pairs were created (96 pairs each of positive-neutral, negative-neutral and positive-negative words). The word pairs were split into 4 separate sets of stimuli as described in the main text, with each set containing 72 pairs (24 each of the three possible valence combinations). The specific words used in the pairs were selected to be identical in length. In addition the pairs of words used in each set were balanced in terms of Kucera-Francis Frequency [all $t(71) < 1.6$, $p > 0.12$].

In order to ensure that tests of attentional bias used novel stimuli, the lists of word and face stimuli were split into four separate sets with each of the three assessment sessions deploying a novel set of stimuli (the sets used were counterbalanced across patients). The stimuli used in ABM were taken from the fourth set combined with the set of stimuli used in the pre-ABM assessment.

Compliance with Bias Modification. Compliance with the bias modification interventions was encouraged by planning with patients when during the day they would complete the task (35). In addition patients were contacted by researchers on two occasions during the treatment fortnight to check that they were complying with the study protocol. The number of times a participant completed ABM was recorded automatically. For the purpose

of analysis, compliance with the ABM regime was defined as completing at least 25 out of a total of 28 sessions.

Cortisol Assay

Salivary cortisol was measured, blind to subject status, by an in-house double-antibody radioimmunoassay with utilization of 125I-cortisol as the ligand. The intra- and inter-assay coefficients of variation were 4.1% and 7.8% respectively and the minimum detectable concentration was 0.2 nmol/L when a 0.1 ml volume was assayed.

Supplementary Analysis

Did the Effects of ABM on Attentional Bias Differ as a Function of Stimuli Valence or Duration?

The analysis reported in the main manuscript described a time x ABM type effect on attentional bias as measured by the word based visual probe. This indicates that the positive and placebo ABM tasks had a differential effect on attentional bias. However the visual probe task included trials with three different pairs of stimuli valence (positive-neutral, positive-negative, negative-neutral) and two different durations (500 ms and 1000 ms) which makes it possible to test for more specific effects of the ABM task. For example, it allows assessment of whether the ABM tasks specifically altered attentional bias to brief (500 ms) as opposed to longer (1000 ms) stimuli presentations. This possibility was tested by calculating individual bias scores for each stimuli pair type and each duration. These were then entered into a repeated measures analysis of variance which included the between subject factors of ABM type (positive, placebo) and ABM stimulus (faces, words) and the within subject factors of time (before ABM, after ABM, follow-up), assessment stimuli valence (positive-neutral, positive-negative, negative-neutral) and assessment stimuli duration (500 ms, 1000 ms). Differential effects of the ABM task on attentional bias would be detected in this analysis as a time x ABM type x assessment stimulus parameter effect. This analysis confirmed the reported time x ABM type effect [$F(2,112) = 3.1$, $p = 0.05$], and found no evidence that this effect was modified by any of the assessment stimulus parameters [all $F < 1$, $p > 0.6$]. This indicates that the ABM procedure produces a generalized alteration of affective bias rather than specifically altering bias to a subset of stimulus valences/durations. This is consistent

with the effects found in a previous study of healthy volunteers which used a similar ABM paradigm (1).

Did the Effects of ABM Depend on Whether Participants Became Aware of the Training Contingency?

The ABM task was originally designed as a method for implicitly modifying attentional bias (24). In other words, bias modification should occur even though participants are unable to report the training contingency of the task (i.e. that during positive ABM the probes replace the positive faces). However, some participants will inevitably notice the contingency while completing the task raising the possibility that the impact of bias modification may be accounted for by demand effects specifically in this “aware” subgroup of participants. In the current study, after completion of the final follow-up session, participants were asked whether they had noticed the training contingency. This question was relevant only to those in the positive ABM groups, as there was no contingency to detect in the placebo groups (and no illusory correlations were reported). Of the 16 participants in the positive face-ABM group only three noticed the training contingency, and of the 16 participants in the positive word-ABM group only 4 noticed the contingency (difference between groups using Fisher’s Exact Test, not significant). The number of participants who detected the contingency was therefore too small to allow a direct statistical comparison between “aware” and “unaware” groups. We therefore assessed whether aware participants exerted a disproportionate effect by rerunning the analyses reported in the main paper after removing participants who were aware of the contingency. The analyses revealed an identical effect of ABM on Beck Depression Inventory [ABM type x ABM stimuli x time; $F(2,98) = 4.8$, $p = 0.01$], trait-Spielberger State-Trait Anxiety Inventory [ABM type x ABM stimuli x time; $F(2,98) = 4.1$, $p = 0.02$] and attentional bias [ABM type x ABM stimuli x time; $F(2,98) = 3.2$, $p = 0.05$] with no change to the results of the post hoc tests. Similarly the overall effect of ABM on Hamilton Rating Scale for Depression scores remained unchanged [ABM type x ABM stimuli x time; $F(2,98) = 3.3$, $p = 0.04$]. The effect of face ABM on cortisol awakening response was now apparent at a trend level [ABM type x time; $F(2,46) = 2.7$, $p = 0.08$]. These results provide robust evidence, at least for the mood outcomes, that the impact of ABM was not dependent on participant awareness and therefore cannot be accounted for demand effects.

Did the Number of Sessions of Bias Modification Completed Influence the Results?

Rerunning all the analyses reported in the main paper with an additional covariate coding for the number sessions of ABM completed did not alter any of the reported results. In addition the number of sessions completed did not significantly predict any of the critical outcomes (i.e. change in Beck Depression Inventory, trait-Spielberger State-Trait Anxiety Inventory or cortisol awakening response during the follow-up period or change in attentional bias during the bias modification period) indicating that the effects of ABM did not depend on high levels of compliance.

Supplementary References

1. Browning M, Grol M, Ly V, Goodwin GM, Holmes EA, Harmer CJ (2011): Using an experimental medicine model to explore combination effects of pharmacological and cognitive interventions for depression and anxiety. *Neuropsychopharmacology* 36:2689-2697.
2. Matsumoto D, Ekman P (1988): *Japanese and Caucasian Facial Expressions of Emotion and Neutral Faces (JACFEE and JACNeuF).* San Francisco: Human Interaction Laboratory, University of California.
3. Lundqvist D, Flykt A, Ohman A (1998): *KDEF.* Stockholm: Karolinska Hospital.
4. Ekman P, Friesen WV (1976): *Pictures of Facial Affect*. Palo Alto, California: Consulting Psychologists Press.
5. Tottenham N, Tanaka JW, Leon AC, McCarry T, Nurse M, Hare TA, *et al.* (2009): The NimStim set of facial expressions: judgments from untrained research participants. *Psychiatry Res* 168:242-249.
6. Anderson N (1968): Likableness ratings of 555 personality trait words. *J Pers Soc Psychol* 9:272–279.
7. Asmundson GJ, Wright KD, Hadjistavropoulos HD (2005): Hypervigilance and attentional fixedness in chronic musculoskeletal pain: consistency of findings across modified stroop and dot-probe tasks. *J Pain* 6:497-506.
8. Afzal M, Potokar JP, Probert CS, Munafo MR (2006): Selective processing of gastrointestinal symptom-related stimuli in irritable bowel syndrome. *Psychosom Med* 68:758-761.

9. Beck JG, Freeman JB, Shipherd JC, Hamblen JL, Lackner JM (2001): Specificity of Stroop interference in patients with pain and PTSD. *J Abnorm Psychol* 110:536-543.
10. Boyer MC, Compas BE, Stanger C, Colletti RB, Konik BS, Morrow SB, *et al.* (2006): Attentional biases to pain and social threat in children with recurrent abdominal pain. *J Pediatr Psychol* 31:209-220.
11. Bryant RA, Harvey AG (1995): Processing threatening information in posttraumatic stress disorder. *J Abnorm Psychol* 104:537-541.
12. Dannlowski U, Kersting A, Lalee-Mentzel J, Donges US, Arolt V, Suslow T (2006): Subliminal affective priming in clinical depression and comorbid anxiety: a longitudinal investigation. *Psychiatry Res* 143:63-75.
13. Dozois DJA (2000): Cognitive organization and information processing in clinical depression: The structure and function of sociotropic schemata. *Psychology*, Vol PhD. Calgary: University of Calgary.
14. Dudley R, O'Brien J, Barnett N, McGuckin L, Britton P (2002): Distinguishing depression from dementia in later life: a pilot study employing the Emotional Stroop task. *Int J Geriatr Psychiatry* 17:48-53.
15. Eilola TM, Havelka J, Sharma D (2007): Emotional activation in the first and second language. *Cogn Emot* 21:1064-1076.
16. Foa EB, Feske U, Murdock TB, Kozak MJ, McCarthy PR (1991): Processing of threat-related information in rape victims. *J Abnorm Psychol* 100:156-162.
17. Grant DM, Beck JG (2006): Attentional biases in social anxiety and dysphoria: does comorbidity make a difference? *J Anxiety Disord* 20:520-529.
18. Green M, Corr P, De Silva L (1999): Impaired color naming of body shape related words in anorexia nervosa: affective valence or associative priming? *Cognit Ther Res* 23:413-422.
19. Henderson CJ, Hagger MS, Orbell S (2007): Does priming a specific illness schema result in an attentional information-processing bias for specific illnesses? *Health Psychol* 26:165-173.
20. Intili R, Tarrier N (1998): Attentional bias in morbid jealousy. *Behav Cogn Psychother* 26:323-338.
21. Karademas EC, Sideridis GD, Kafetsios K (2008): Health-related information processing and recent health problems: evidence from a modified stroop task. *J Health Psychol* 13:28-38.

22. Keogh E, Ellery D, Hunt C, Hannent I (2001): Selective attentional bias for pain-related stimuli amongst pain fearful individuals. *Pain* 91:91-100.
23. Kyrios M, Iob MA (1998): Automatic and strategic processing in obsessive-compulsive disorder: attentional bias, cognitive avoidance or more complex phenomena? *J Anxiety Disord* 12:271-292.
24. MacLeod CM, Rutherford E, Campbell L, Ebsworthy G, Holker L (2002): Selective attention and emotional vulnerability: Assessing the causal basis of their association through the experimental manipulation of attentional bias. *J Abnorm Psychol* 111:107-123.
25. Mathews A, Mogg K, May J, Eysenck M (1989): Implicit and explicit memory bias in anxiety. *J Abnorm Psychol* 98:236-240.
26. Lim SL, Kim JH (2005): Cognitive processing of emotional information in depression, panic, and somatoform disorder. *J Abnorm Psychol* 114:50-61.
27. Mattia JI, Heimberg RG, Hope DA (1993): The revised Stroop color-naming task in social phobics. *Behav Res Ther* 31:305-313.
28. McNally RJ, Kaspi SP, Riemann BC, Zeitlin SB (1990): Selective processing of threat cues in posttraumatic stress disorder. *J Abnorm Psychol* 99:398-402.
29. Moss-Morris R, Petrie KJ (2003): Experimental evidence for interpretive but not attention biases towards somatic information in patients with chronic fatigue syndrome. *Br J Health Psychol* 8:195-208.
30. Munafo MR, Hayward G, Harmer C (2006): Selective processing of social threat cues following acute tryptophan depletion. *J Psychopharmacol* 20:33-39.
31. Russo R, Whittuck D, Roberson D, Dutton K, Georgiou G, Fox E (2006): Mood-congruent free recall bias in anxious individuals is not a consequence of response bias. *Memory* 14:393-399.
32. Taghavi MR, Dalgleish T, Moradi AR, Neshat-Doost HT, Yule W (2003): Selective processing of negative emotional information in children and adolescents with Generalized Anxiety Disorder. *Br J Clin Psychol* 42:221-230.
33. Thrasher SM, Dalgleish T, Yule W (1994): Information processing in post-traumatic stress disorder. *Behav Res Ther* 32:247-254.
34. van Hooff JC, Dietz KC, Sharma D, Bowman H (2008): Neural correlates of intrusion of emotion words in a modified Stroop task. *Int J Psychophysiol* 67:23-34.

35. Blackwell SE, Holmes EA (2010): Modifying interpretation and imagination in clinical depression: a single case series using cognitive bias modification. *Appl Cogn Psychol* 24:338-350.
